# Blood pressure, hypertension and the risk of sudden cardiac death: a systematic review and meta-analysis of cohort studies

**DOI:** 10.1007/s10654-019-00593-4

**Published:** 2019-12-24

**Authors:** Han Pan, Makoto Hibino, Elsa Kobeissi, Dagfinn Aune

**Affiliations:** 1 Department of Epidemiology and Biostatistics, School of Public Health, Imperial College London, St. Mary’s Campus, Norfolk Place, Paddington, London, W2 1PG UK; 2 Department of Nutrition, Bjørknes University College, Oslo, Norway; 3 Department of Endocrinology, Morbid Obesity and Preventive Medicine, Oslo University Hospital, Oslo, Norway

**Keywords:** Blood pressure, Hypertension, Death, sudden, cardiac, Systematic review, Meta-analysis, Cohort studies

## Abstract

Cardiovascular disease is the leading cause of death worldwide, while sudden cardiac death (SCD) accounts for over 60% of all cardiovascular deaths. Elevated blood pressure and hypertension have been associated with increased risk of SCD, but the findings have not been consistent. To clarify whether blood pressure or hypertension is associated with increased risk of SCD and to quantify the size and the shape of any association observed. PubMed and Embase databases were searched for published prospective studies on blood pressure or hypertension and SCD up to 30 April 2018. Summary relative risks (RRs) and 95% confidence intervals (CIs) were calculated using a random effects model. The meta-analysis included 2939 SCDs among 418,235 participants from 18 studies. The summary RRs were 2.10 (95% CI 1.71–2.58, I^2^ = 56.7%, p_heterogeneity_ = 0.018, n = 10) for prevalent hypertension, 1.28 (95% CI 1.19–1.38, I^2^ = 45.5%, p_heterogeneity_ = 0.07, n = 10) per 20 mmHg increment in systolic blood pressure (SBP) and 1.09 (95% CI 0.83–1.44, I^2^ = 83.4%, p_heterogeneity_ = 0.002, n = 3) per 10 mmHg increment in diastolic blood pressure (DBP). A nonlinear relationship was suggested between SBP and SCD. The results persisted in most subgroup and sensitivity analyses. There was no evidence of publication bias. This meta-analysis found an increased risk of SCD with hypertension diagnosis and increasing SBP. Future studies should clarify the association for DBP and the shape of the dose–response relationship between blood pressure and SCD.

**Electronic supplementary material:**

The online version of this article (10.1007/s10654-019-00593-4) contains supplementary material, which is available to authorized users.

## Introduction

Cardiovascular disease (CVD) is a major cause of morbidity and mortality worldwide [1, 2]. Sudden cardiac death (SCD)—the fatal event following sudden cardiac arrest (SCA)—accounts for more than 60% of all cardiovascular deaths [3]. Despite advances in cardiopulmonary resuscitation and post-resuscitation management, the prognosis and outcomes following SCA remain very poor [3–5]. The average survival rate of SCA ranges between 5 and 10% in developed regions, whereas the estimated global survival rate of SCA is less than 1% [3–5]. Most of SCA/SCD cases arise in the general population [3, 6, 7]. Approximately half of SCDs are the first manifestation of CVDs [3]. Primary prevention using population-wide strategies is therefore of great importance in reducing the burden of SCD.

Elevated blood pressure is the single largest contributor to the global burden of disease, accounting for two-thirds of strokes, half of coronary heart disease (CHD) cases and a total of 9.4 million global deaths per year [8–10]. Several cohort studies have been published on the association between hypertension and the risk of SCD [6, 11–14]; and all but one [14] found a positive association between the two. However, the available studies differed considerably with regard to the strength of the associations reported with relative risks (RRs) ranging between 1.4 and 3.82 [6, 11–14], indicating a risk elevation of 40% up to nearly four folds. Studies have also examined the relationship between blood pressure and the risk of SCD [15–18]. Most of these reported an increase in risk with higher blood pressure [15–17], although one found no significant association [18]; but again the reported RRs differed considerably regarding the strength of the association [15–17]. Therefore, we conducted a systematic review and meta-analysis of cohort studies on the association between hypertension or blood pressure and the risk of SCD with an aim of clarifying the presence and strength of the association as well as to investigate the dose–response relationship and potential sources of heterogeneity in the results.

## Methods

The study was conducted according to the preferred reporting items for systematic reviews and meta-analyses (PRISMA) statement [19]. The protocol has been registered on PROSPERO (CRD42018096736).

### Search strategy

PubMed and Embase were searched from inception up to 30 April 2018. The search terms used are shown in Supplementary Table 1. The reference lists of the included publications were also screened with additional studies being identified.

### Inclusion criteria and study selection

Prospective cohort studies, retrospective cohort studies, nested case–control studies and case-cohort studies of general adult populations that provide risk estimates for the association between blood pressure or hypertension and the risk of SCD with adjustment for at least one confounding factor were included. When multiple articles had been published on the same exposure from the same study, the one with the largest number of participants and/or cases or adjusted for SCD risk factors with higher level of evidence was chosen.

### Data extraction

The following data were extracted from each included study: name of the first author, study publication year, country where the study was conducted, name of the study, study period and the length of follow-up, sample size and participant characteristics (sex, age, occupation and baseline disease status), number of cases, type of exposure (hypertension, antihypertensive medication use, systolic blood pressure (SBP) or diastolic blood pressure (DBP)), subgroup(s), RRs and 95% confidence intervals (CIs) and confounder(s) adjusted for in the analysis. For studies in which RRs and 95% CIs were not readily available, conversions were made using listed regression coefficients, standard errors, p values and/or t/z values.

### Quality assessment

Study quality was evaluated using the Newcastle–Ottawa scale [20]. ‘Demonstration that outcome of interest was not present at start of study’ was defined as exclusion of participants with prevalent CHD at baseline. Follow-up of 5 years or longer and lost to follow-up rate of 10% or less were considered as adequate follow-up period and percentage, respectively. The total score has a range from 0 to 9.

### Statistical methods

Random effects models [21] which account for both within- and between-study variance were applied to assess the association between blood pressure or hypertension and SCD. A *p* value < 0.05 was considered statistically significant. The method by Greenland and Longnecker [22] was adopted for the linear dose–response analyses of blood pressure and SCD. Linear trends were generated using the natural logarithms of the reported RRs and 95% CIs of each blood pressure category. Studies with SBP and/or DBP as continuous variables first underwent unit conversion to give risk estimates for blood pressure categories. Midpoints for each category were calculated as the average of the upper and lower cut-off values. For open-ended categories, the width of the adjacent category was used to calculate the upper and/or lower cut-off points. Results for studies which only reported risk estimates stratified by sex or age, but not overall, were pooled using the fixed effect model before inclusion in the meta-analysis. Fractional polynomial models [23] were used for the nonlinear dose–response analysis of blood pressure and SCD. The best fitting second order fractional polynomial regression model was defined as the one with the lowest deviance [23]. Nonlinearity was evaluated by a likelihood ratio test comparing the nonlinear and linear models [23].

Heterogeneity between studies was assessed using the Q statistic and the I^2^ statistic [24]. I^2^ values of approximately 25%, 50% and 75% correspond to low, moderate and high degrees of heterogeneity, respectively. Subgroup and sensitivity analyses were conducted to evaluate the result consistency across various study characteristics and to investigate sources of heterogeneity between studies. Pre-specified factors included: sex, publication year, length of follow-up, geographic location, number of cases, study quality, baseline CHD status, hypertension definition, SCD definition, age and other adjusted confounders. Meta-regression analyses were used to test for differences between subgroup analyses and to examine the impact of individual characteristics on the overall effect size and/or their contributions to the detected heterogeneity [20]. Sensitivity analyses excluding one study at a time to evaluate the impact of individual studies on the overall results were also conducted. Publication bias was assessed by Egger’s test [25], Begg’s test [26] and by inspection of the funnel plots. A *p* value < 0.10 was considered as an indication of potential publication bias. All statistical analyses were completed using the Stata software, version 13.1 (StataCorp, Texas, US).

## Results

### Study selection

**Fig. 1:**
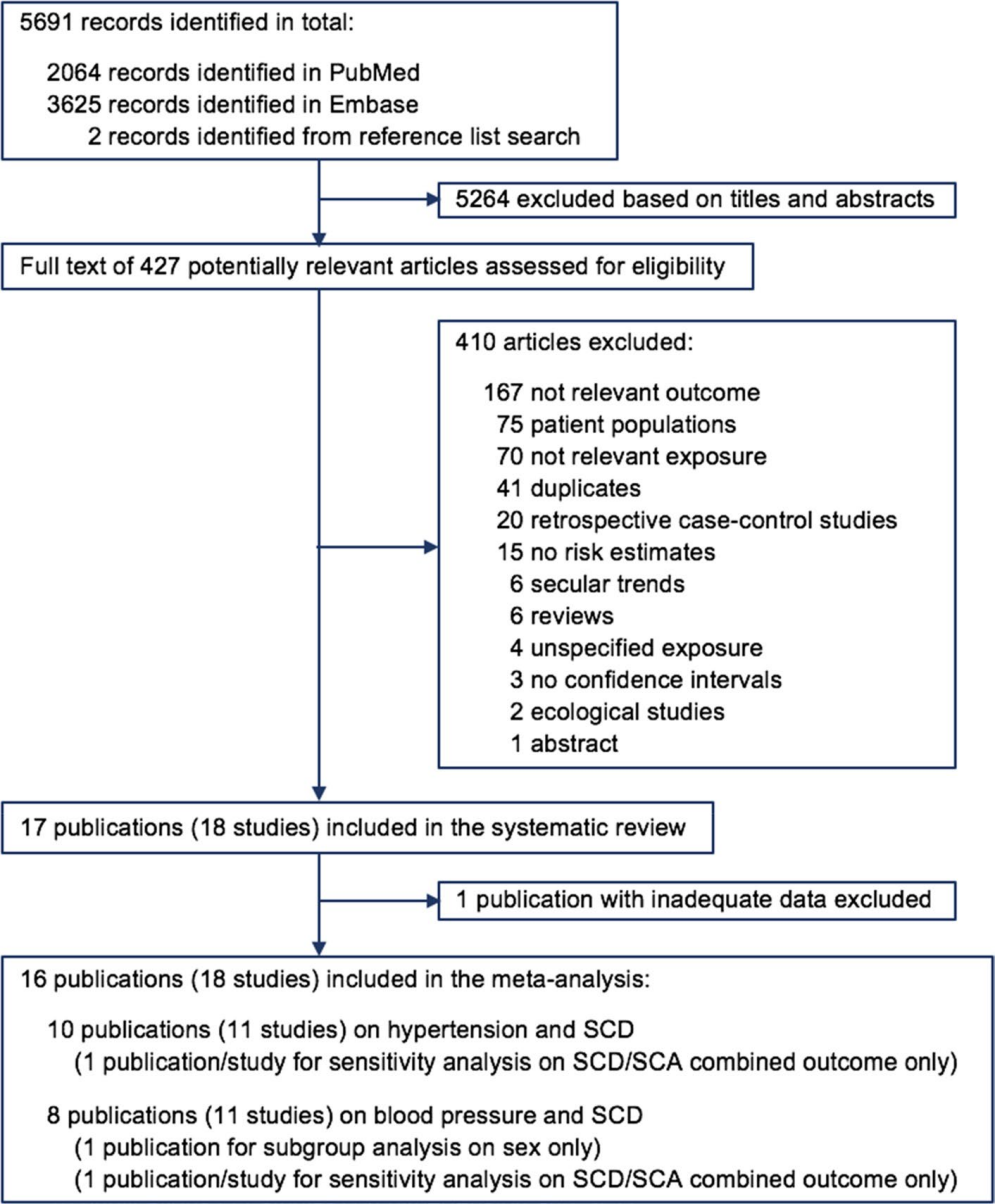
Flow diagram of study selection for the systematic review and meta-analysis of cohort studies on blood pressure, hypertension and the risk of sudden cardiac death (SCD, sudden cardiac death; SCA, sudden cardiac arrest)

A total of 5691 records were screened, of which 5689 records were identified from database search and two additional studies [15, 27] were identified by reference list searching. Based on titles and abstracts, 5264 records were excluded. The full texts of the remaining 427 articles were carefully examined. Among them, 397 were further excluded for reasons listed in Fig. 1. After removing the duplicates of the two databases, 18 studies (17 publications) [6, 7, 11–13, 15–18, 27–34] remained. One publication with inadequate data (insufficient blood pressure categories for data analysis) [16] was further excluded from the meta-analysis. Two publications [27, 34] were only used in subgroup and sensitivity analyses – one [27] overlapped with another publication [15] which was used for the main analysis and the other [34] assessed a combined outcome of SCA/SCD thus did not meet the eligibility criteria for the primary analysis.

### Study characteristics

Detailed study-level characteristics and the extracted data are presented in Supplementary Table 2 and Supplementary Table 3 for studies on hypertension and blood pressure, respectively. One study was a nested case–control study [11] and all the other studies were prospective cohort studies. Eleven studies were conducted in Europe [13, 16–18, 29–34], six were from the United States [6, 7, 12, 15, 27, 28] and one was from Japan [11]. Six studies recruited participants free of baseline CHD or reported risk estimates for people without prior CHD [6, 15, 26, 27, 29, 34]. The length of follow-up ranged from 3.5 to 26 years.

### Hypertension and the risk of SCD

**Fig. 2:**
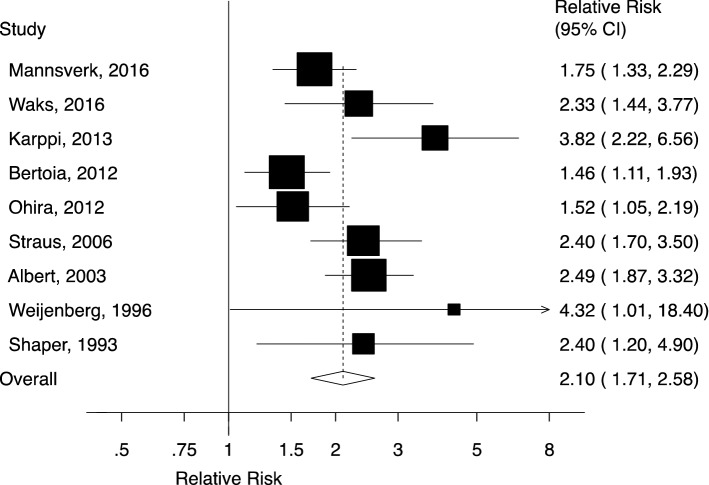
Forest plot of the meta-analysis of cohort studies on hypertension and sudden cardiac death (CI, confidence interval)

Ten studies (nine publications) [6, 11–13, 28–31, 33] involving 1991 SCDs among 372,484 participants were included in the meta-analysis of hypertension and the risk of SCD (Fig. 2). Eight studies assessed hypertension [6, 11, 12, 28, 29, 31, 33], and the two remaining studies evaluated antihypertensive use [13, 30]. The summary RR of SCD for patients with hypertension versus participants without hypertension was 2.10 (95% CI 1.71–2.58, I^2^ = 56.7%, p_heterogeneity_ = 0.02). There was neither evidence of publication bias with Begg’s test (*p* = 0.47) or Egger’s test (*p* = 0.14), nor asymmetry indicated by the funnel plot (Supplementary Fig. 1). When individual studies were excluded from the analysis one at a time, the summary RRs ranged from 1.96 (95% CI 1.63–2.36) when the study by Karppi et al. [13] was excluded to 2.23 (95% CI 1.82–2.73) when the study by Bertoia et al. [6] was excluded (Supplementary Fig. 2).

### Blood pressure and the risk of SCD

The meta-analysis of blood pressure and the risk of SCD was comprised of 11 studies (seven publications) [7, 15–18, 30, 32]. One publication contained data from four studies [18].

**Fig. 3:**
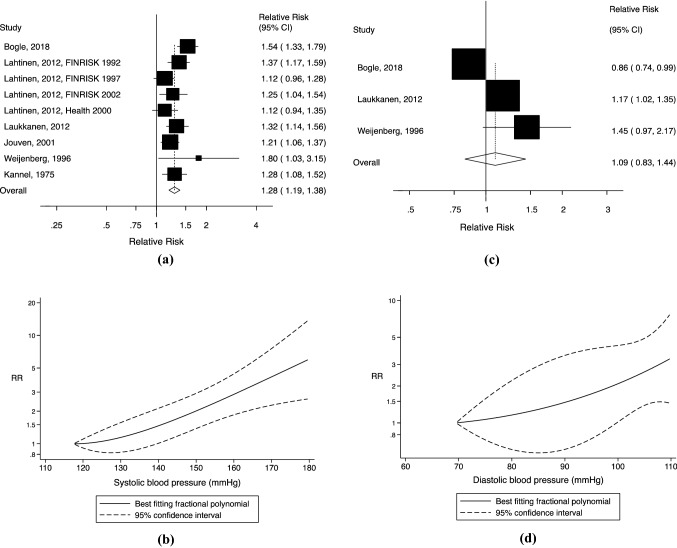
**a** Forest plot of the meta-analysis of cohort studies on systolic blood pressure (per 20 mmHg) and sudden cardiac death; **b** Nonlinear dose–response analysis of cohort studies on systolic blood pressure and sudden cardiac death. **c** Forest plot of the meta-analysis of cohort studies on diastolic blood pressure (per 10 mmHg) and sudden cardiac death; **d** Nonlinear dose–response analysis of cohort studies on diastolic blood pressure and sudden cardiac death (CI, confidence interval; RR, relative risk)

The linear dose–response analysis of SBP and SCD included ten studies (six publications) [7, 15, 17, 18, 30, 32] with 1123 SCDs among 57,494 participants (Fig. 3a). The summary RR of SCD was 1.28 (95% CI 1.19–1.38, I^2^ = 45.5%, p_heterogeneity_ = 0.07) per 20 mmHg increment of SBP. Begg’s test (*p* = 0.92), Egger’s test (*p* = 0.50) and the inspection of the funnel plot (Supplementary Fig. 3) suggested no evidence of publication bias. The influence analysis presented a range of summary RRs from 1.24 (95% CI 1.17–1.32) when the study by Bogle et al. [7] was excluded to 1.31 (95% CI 1.21–1.41) when the study by Lahtinen et al. (FINRISK 1997) [18] was excluded (Supplementary Fig. 4). There was evidence of a nonlinear relationship between SBP and SCD (p_nonlinearity_ < 0.001), with a slightly steeper increase in risk from around 140 mmHg (Fig. 3b, Supplementary Table 4). However, only two small male studies^30,32^ were included in the analysis.

The linear dose–response analysis of DBP and SCD consisted of three studies/publications [7, 30, 32] and included 402 SCDs among 18,666 participants (Fig. 3c). The summary RR for a 10 mmHg increment of DBP was 1.09 (95% CI 0.83–1.44, I^2^ = 83.4%, p_heterogeneity_ = 0.002). No evidence of publication bias was indicated (Begg’s test *p* = 0.99, Egger’s test *p* = 0.71, funnel plot shown in Supplementary Fig. 5). There was no evidence of a nonlinear association between DBP and SCD (p_nonlinearity_ = 0.67, Fig. 3d, Supplementary Table 5); but again, the results were based on two relatively small male studies [30, 32].

### Quality assessment

The overall study quality was high with a mean (median) of 7.7 (8) out of 9 across all included studies (Supplementary Table 6).

### Subgroup and sensitivity analyses

The subgroup and sensitivity analyses results are shown in Table 1. For hypertension and SBP, which displayed significantly increased risks for SCD in the primary analyses, nearly all subgroup analyses maintained the positive association with statistical significance. The subgroup analyses of DBP and SCD were limited by the few studies. When studies on hypertension were stratified by adjustments for resting heart rate and atrial fibrillation (AF), the summary RR was much weaker among the studies with such adjustments than among those without (RR = 1.48, 95% CI 1.19–1.85 vs. RR = 2.35, 95% CI 1.94–2.86; p_heterogeneity_ = 0.03). No significant heterogeneity was present between the remaining subgroups. Nevertheless, most analyses showed substantial heterogeneity within each subgroup. One exception was male-only studies, which had zero heterogeneity across all types of exposure.

**Table 1:** Subgroup analyses of hypertension or blood pressure and sudden cardiac death in cohort studies

	Hypertension				
	n	RR (95% CI)	I^2^ (%)	P_h_^a^	P_h_^b^
All studies	10	2.10 (1.71–2.58)	56.7	0.02	
Sex					
Men	3	3.29 (2.18–4.97)	0	0.55	0.18/0.23^c^
Women	2	1.90 (1.13–3.21)	85.5	0.01	
Men and women	5	1.91 (1.55–2.35)	26.1	0.26	
Publication year					
Before 2010	4	2.48 (2.01–3.06)	0	0.90	0.19
2010 onwards	6	1.91 (1.45–2.52)	65.3	0.02	
Length of follow-up					
< 10 years	4	2.06 (1.49–2.86)	32.7	0.22	0.96
≥ 10 years	6	2.13 (1.60–2.85)	71.4	0.01	
Geographic location					
Europe	5	2.42 (1.77–3.31)	48.9	0.10	0.20
America	4	2.01 (1.38–2.92)	73.4	0.02	
Asia	1	1.52 (1.05–2.20)			
Number of cases					
< 250	6	2.39 (1.83–3.10)	46.4	0.10	0.15
≥ 250	4	1.71 (1.37–2.14)	29.6	0.24	
Study quality					
0–3 stars	0				0.72
4–6 stars	2	1.97 (1.22–3.20)	76.8	0.04	
7–9 stars	8	2.16 (1.67–2.80)	57.2	0.03	
Antihypertensive medication use as a proxy for hypertension diagnosis					
Yes	2	3.88 (2.33–6.44)	0	0.88	0.06
No	8	1.94 (1.61–2.33)	47.3	0.08	
Hypertension definition					
≥ 140/90 mmHg	6	1.84 (1.48–2.29)	55.7	0.06	0.31
≥ 160/90 mmHg^d^	2	2.40 (1.74–3.31)	0	0.99	
Sudden cardiac death definition with strict 1-h criterion^e^					
Yes	3	1.99 (1.32–3.01)	72.6	0.03	0.68
No	7	2.18 (1.67–2.86)	54.3	0.05	
Participants free of baseline coronary heart disease					
Yes	3	1.71 (1.40–2.09)	0	0.53	0.14
No	7	2.37 (1.85–3.04)	46.3	0.10	
Adjustment for confounding factors					
Age					
Yes	10	2.10 (1.71–2.58)	56.7	0.02	NC
No	0				
Race					
Yes	3	1.77 (1.13–2.77)	63.2	0.10	0.35
No	7	2.23 (1.77–2.81)	51.2	0.06	
Geographic region					
Yes	1	1.52 (1.05–2.20)			0.28
No	9	2.20 (1.76–2.75)	56.6	0.02	
Family history of cardiovascular diseases					
Yes	1	2.49 (1.87–3.32)			0.55
No	9	2.04 (1.62–2.56)	55.1	0.03	
Body mass index/weight					
Yes	4	1.86 (1.31–2.64)	67.8	0.03	0.30
No	6	2.32 (1.79–3.01)	43.2	0.13	
Waist					
Yes	2	2.29 (0.90–5.88)	89.6	0.002	0.97
No	8	2.08 (1.75–2.48)	23.1	0.25	
Physical activity					
Yes	1	2.40 (1.19–4.85)			0.77
No	9	2.09 (1.67–2.60)	61.5	0.01	
Smoking					
Yes	6	2.17 (1.56–3.02)	69.0	0.01	0.96
No	4	2.04 (1.64–2.53)	12.4	0.32	
Alcohol consumption					
Yes	4	2.50 (1.45–4.31)	65.6	0.03	0.53
No	6	2.00 (1.60–2.49)	57.4	0.05	
Resting heart rate					
Yes	2	1.48 (1.19–1.85)	0	0.86	0.03
No	8	2.35 (1.94–2.86)	28.1	0.21	
Blood glucose					
Yes	1	2.40 (1.19–4.85)			0.77
No	9	2.09 (1.67–2.60)	61.5	0.01	
Serum cholesterol					
Yes	3	3.29 (2.18–4.97)	0	0.55	0.08
No	7	1.92 (1.57–2.34)	54.4	0.05	
Diabetes mellitus					
Yes	6	2.10 (1.52–2.90)	73.7	0.004	0.85
No	4	2.05 (1.64–2.56)	7.1	0.36	
Adjustment for potential intermediate risk factors					
Coronary heart disease including myocardial infarction					
Yes	5	2.29 (1.56–3.38)	76.6	0.01	0.59
No	5	1.92 (1.55–2.38)	20.5	0.28	
Heart failure					
Yes	2	2.29 (0.90–5.88)	89.6	0.002	0.97
No	8	2.08 (1.75–2.48)	23.1	0.25	
Atrial fibrillation					
Yes	2	1.48 (1.19–1.85)	0	0.86	0.03
No	8	2.35 (1.94–2.86)	28.1	0.21	

	Systolic blood pressure (per 20 mmHg)					Diastolic blood pressure (per 10 mmHg)				
	n	RR (95% CI)	I^2^ (%)	P_h_^a^	P_h_^b^	n	RR (95% CI)	I^2^ (%)	P_h_^a^	P_h_^b^
All studies	10	1.28 (1.19–1.38)	45.5	0.07		3	1.09 (0.83–1.44)	83.4	0.002	
Sex										
Men	4	1.26 (1.16–1.38)	0	0.51	0.95/0.29^c^	2	1.20 (1.05–1.37)	0	0.32	0.19
Women	1	1.04 (0.78–1.38)				0				
Men and women	5	1.28 (1.12–1.45)	67.6	0.02		1	0.86 (0.74–0.99)			
Publication year										
Before 2010	4	1.25 (1.13–1.38)	0	0.38	0.92	1	1.45 (0.97–2.17)			0.46
2010 onwards	6	1.29 (1.16–1.43)	59.9	0.03		2	1.00 (0.74–1.36)	89.0	0.003	
Length of follow-up										
< 10 years	3	1.22 (1.04–1.44)	25.9	0.26	0.56	1	1.45 (0.97–2.17)			0.46
≥ 10 years	7	1.30 (1.19–1.42)	54.8	0.05		2	1.00 (0.74–1.36)	89.0	0.003	
Geographic location										
Europe	7	1.24 (1.15–1.33)	15.0	0.32	0.12	2	1.20 (1.05–1.37)	0	0.32	0.19
America	3	1.42 (1.18–1.70)	60.9	0.11		1	0.88 (0.74–0.99)			
Number of cases										
< 150	7	1.25 (1.17–1.35)	0	0.46	0.62	1	1.45 (0.97–2.17)			0.46
≥ 150	3	1.32 (1.10–1.59)	78.6	0.01		2	1.00 (0.74–1.36)	89.0	0.003	
Study quality										
0–3 stars	0				NC	0				NC
4–6 stars	0					0				
7–9 stars	10	1.28 (1.19–1.38)	45.5	0.07		3	1.09 (0.83–1.44)	83.4	0.002	
Sudden cardiac death definition with strict 1-h criterion^e^										
Yes	4	1.34 (1.15–1.56)	68.1	0.04	0.43	1	0.86 (0.74–0.99)			0.19
No	6	1.25 (1.14–1.36)	27.9	0.23		2	1.20 (1.05–1.37)	0	0.32	
Participants free of baseline coronary heart disease										
Yes	3	1.24 (1.12–1.37)	0	0.59	0.66	0				NC
No	7	1.30 (1.17–1.44)	56.5	0.03		3	1.09 (0.83–1.44)	83.4	0.002	
Adjustment for confounding factors										
Age										
Yes	10	1.28 (1.19–1.38)	45.5	0.07	NC	3	1.09 (0.83–1.44)	83.4	0.002	NC
No	0					0				
Geographic region										
Yes	4	1.21 (1.10–1.34)	30.1	0.23	0.21	0				NC
No	6	1.35 (1.21–1.50)	45.6	0.12		3	1.09 (0.83–1.44)	83.4	0.002	
Family history of cardiovascular diseases										
Yes	2	1.25 (1.14–1.38)	0	0.39	0.81	1	1.17 (1.02–1.35)			0.88
No	8	1.29 (1.16–1.43)	56.3	0.03		2	1.08 (0.64–1.80)	83.1	0.02	
Body mass index/weight										
Yes	10	1.28 (1.19–1.38)	45.5	0.07	NC	3	1.09 (0.83–1.44)	83.4	0.002	NC
No	0					0				
Physical activity										
Yes	5	1.21 (1.13–1.30)	6.8	0.37	0.06	0				NC
No	5	1.40 (1.26–1.56)	24.4	0.27		3	1.09 (0.83–1.44)	83.4	0.002	
Smoking										
Yes	10	1.28 (1.19–1.38)	45.5	0.07	NC	3	1.09 (0.83–1.44)	83.4	0.002	NC
No	0					0				
Alcohol consumption										
Yes	2	1.36 (1.14–1.63)	6.1	0.30	0.50	2	1.20 (1.05–1.37)	0	0.32	0.19
No	8	1.27 (1.16–1.38)	53.9	0.04		1	0.86 (0.74–0.99)			
Resting heart rate										
Yes	1	1.21 (1.06–1.38)			0.58	0				NC
No	9	1.30 (1.19–1.41)	49.3	0.06		3	1.09 (0.83–1.44)	83.4	0.002	
Serum cholesterol										
Yes	10	1.28 (1.19–1.38)	45.5	0.07	NC	3	1.09 (0.83–1.44)	83.4	0.002	NC
No	0					0				
Diabetes mellitus										
Yes	7	1.27 (1.17–1.39)	54.7	0.04	0.65	2	1.00 (0.74–1.36)	89.0	0.003	0.46
No	3	1.36 (1.06–1.74)	20.5	0.26		1	1.45 (0.97–2.17)			
QT-prolonging medication										
Yes	3	1.15 (1.05–1.27)	0	0.63	0.06	0				NC
No	7	1.35 (1.24–1.47)	32.8	0.19		3	1.09 (0.83–1.44)	83.4	0.002	
Digoxin										
Yes	3	1.15 (1.05–1.27)	0	0.63	0.06	0				NC
No	7	1.35 (1.24–1.47)	32.8	0.19		3	1.09 (0.83–1.44)	83.4	0.002	
Antihypertensive medication										
Yes	2	1.43 (1.23–1.67)	50.6	0.16	0.07	2	1.00 (0.74–1.36)	89.0	0.003	0.46
No	8	1.23 (1.15–1.31)	7.4	0.37		1	1.45 (0.97–2.17)			
Adjustment for potential intermediate risk factors										
Coronary heart disease including myocardial infarction										
Yes	5	1.24 (1.14–1.34)	23.8	0.26	0.29	1	1.17 (1.02–1.35)			0.88
No	5	1.36 (1.18–1.57)	59.1	0.06		2	1.08 (0.64–1.80)	83.1	0.02	
Left ventricular hypertrophy										
Yes	3	1.31 (1.16–1.46)	0	0.81	0.87	1	1.17 (1.02–1.35)			0.88
No	7	1.28 (1.15–1.41)	58.5	0.03		2	1.08 (0.64–1.80)	83.1	0.02	

*n* number of studies; *RR* relative risk; *CI* confidence interval; *NC* not calculable due to lack of studies in one of the subgroups

^a^P for within-subgroup heterogeneity

^b^P for between-subgroup heterogeneity generated from meta-regression analysis

^c^P for men-versus-women heterogeneity generated from meta-regression analysis (studies with both sexes excluded)

^d^Cut-offs used by the two studies for hypertension diagnosis were 160/90 mmHg and 160/100 mmHg, respectively

^e^Death occurred within 1 h of the onset of symptoms

A sensitivity analysis was conducted to incorporate one study with a combined outcome of SCA/SCD [34], including a total of 5294 SCA/SCD cases among 1676,241 participants. The summary RRs were 2.00 (95% CI 1.52–2.63, I^2^ = 88.0%, p_heterogeneity_ < 0.001) for prevalent hypertension and 1.25 (95% CI 1.15–1.36, I^2^ = 63.9%, p_heterogeneity_ = 0.003) and 1.08 (95% CI 0.92–1.27, I^2^ = 77.4%, p_heterogeneity_ = 0.004) per 20 mmHg and 10 mmHg increments in SBP and DBP, respectively. There was no change to the direction or the significance of the associations.

## Discussion

This is, to the best of our knowledge, the first systematic review and meta-analysis of cohort studies on blood pressure, hypertension and the risk of SCD. The results suggested a twofold increase in risk for SCD with prevalent hypertension and a 28% increase in risk for SCD per 20 mmHg increment in SBP, while no significant association was established between DBP and SCD. The results were robust regarding the association between hypertension/SBP and SCD, whereas the analysis for DBP and SCD was limited by the few studies included. Additionally, there was evidence of a nonlinear relationship between SBP, but not DBP, and SCD, with a slightly steeper association at higher levels. Although the nonlinear analysis contained only two small male studies, there was a clear dose–response relationship. Overall, the analyses involved good-to-high-quality studies from three continents with close to 3000 SCDs among a total of more than 410,000 participants. There was no evidence of publication bias; and the relationships were also maintained in most subgroup and sensitivity analyses.

The findings are partly consistent with a pooled analysis of 61 prospective studies (553 sudden deaths, 1 million participants) [10], which found a reduced risk of sudden death with lower SBP. However, the association was much weaker in the current analysis with a 28% increased risk per 20 mmHg SBP compared to a 104% increased risk for the same increment in the pooled analysis. The exact reason for the difference is unclear—whether some of the studies in the current meta-analysis have over-adjusted by including intermediate risk factors in the multivariable models, or the current meta-analysis provided more conservative and stable risk estimates by including more cases (1123 SCDs vs. 553 sudden deaths), or if there are other contributing factors.

The risk estimates differed substantially when the studies were stratified by adjustments for resting heart rate and AF, which is consistent with previously published systematic reviews and meta-analyses that established positive relationships between resting heart rate/AF and SCD [35, 36]. Nonetheless, whether the difference is due to adjusted confounders being intermediate factors or other characteristics of the studies is not clear, as ideally one would examine the impact of such adjustments within the same study. Although there was high heterogeneity in several analyses, there was no evidence of heterogeneity among male-only studies across all types of exposure, and all suggested a significantly increased risk for SCD. The subgroup analysis by sex for studies on hypertension showed a higher risk for men than for women, though the difference did not reach statistical significance. These observations could indicate sex differences in SCD [3, 4, 9]. However, the number of studies involved in the analyses was rather limited, and caution should be made when interpreting the findings.

Several potential mechanisms could explain an association between high blood pressure or hypertension and increased risk of SCD. First, chronic elevated blood pressure induces adaptive myocardial structural changes, which in turn leads to a cascade of anatomical and functional changes causing left ventricular hypertrophy (LVH) [37]. LVH is an independent risk factor for ventricular arrhythmia [37] and is associated with a 2.5-fold increased risk for SCD [38]. Second, elevated blood pressure increases the risk for CHD [9, 10], with the latter being associated with a fourfold increased risk for SCD [18]. Third, hypertension accounts for nearly 40% and 60% of all heart failure (HF) cases in men and women, respectively [39]. Studies have shown that the presence of HF increases the risk of SCD by as much as fivefold [3]. Lastly, hypertension causes electrophysiological and structural changes in the left atrium that could alter the size and function of the left atrium, acting as a key factor to the pathogenesis of AF [40]. Previous meta-analysis results have demonstrated that AF carries significantly increased risk for SCD in the general population and particular patient groups with a pooled risk ratio of 2.0 [36]. Nevertheless, subgroup analyses conducted in the current study suggest that the increased risk of SCD is independent of most of the above-mentioned factors. However, the association was weaker among studies that adjusted for AF (summary RR = 1.48) compared to studies with no such adjustment (summary RR = 2.35), but the limited number of studies in the subgroup with AF adjustment make firm conclusions difficult.

Limitations of the current meta-analysis include potential confounding, exposure and/or outcome misclassification and between-study heterogeneity. Although only studies with adjusted RRs were included and in-depth analyses suggested little heterogeneity among subgroups, residual confounding cannot be entirely ruled out. Some studies may have also inappropriately adjusted for intermediate factors on the causal pathway from elevated blood pressure to SCD, such as LVH, CHD, HF or AF.

Most studies defined hypertension based on blood pressure measurements and antihypertensive use. Medication use was mainly self-reported, which may have under-estimated the actual number of people with hypertension and the RRs. A few studies also adopted higher blood pressure cut-off values for hypertension diagnosis. However, the sensitivity analysis suggested no significant heterogeneity among studies with different hypertension definitions. Moreover, SCD case adjudication has always been challenging due to often unwitnessed deaths and/or inadequate information [3]. The included studies applied different SCD criteria, which may have over- or under-estimated the incidence of SCDs. The subgroup and sensitivity analyses showed no significant differences between studies that strictly defined SCD as death occurring within 1 h of symptom onset and those with more inclusive criteria. However, more detailed analysis was not feasible due to the varying definitions used by different studies.

There was considerable between-study heterogeneity as well. Nevertheless, except for the analyses on DBP which only included three studies, the reported heterogeneity was mainly due to differences in the size of the association rather than the overall direction.

This systematic review and meta-analysis has a number of strengths. The inclusion of cohort studies ensured that the exposure preceded the outcome, avoided recall bias and reduced the potential for selection bias. The meta-analysis included close to 3000 SCDs in over 410,000 participants, providing sufficient statistical power to detect even moderate associations between hypertension/SBP and SCD. The high quality of the included studies and the detailed subgroup and sensitivity analyses further ascertained the robustness of the results. Finally, the dose–response analysis provided additional evidence for a dose–response relationship between elevated blood pressure and the risk of SCD.

Further studies should aim to clarify the association between DBP and SCD and the shape of the dose–response relationship between blood pressure and SCD. As most included studies were from Europe and North America, additional studies are needed to clarify these associations in other geographic locations and among different ethnicities. Only studies from the general population were included in this systematic review and meta-analysis. Further studies could clarify whether modifying factors such as the use of drug therapies and its related biochemical and/or physiological influences would affect the association between hypertension or blood pressure and SCD. Given that hypertension is linked to various lifestyle factors, future studies should also address the association between lifestyle components and SCD directly, as it is possible that elevated blood pressure might be a mediator of the adverse effects of other risk factors. Public health policies and interventions to further address the hypertension epidemic are recommended to reduce the burden of SCD.

## Electronic supplementary material

Below is the link to the electronic supplementary material.

Supplementary material 1 (DOCX 696 kb)
