# Supplementary material for: Blood pressure, hypertension and the risk of sudden cardiac death: a systematic review and meta-analysis of cohort studies
Source: Eur J Epidemiol. 2019 Dec 24;35(5):443–54. doi: 10.1007/s10654-019-00593-4 (PMC7250808; doi:10.1007/s10654-019-00593-4)
Supplement: Supplementary file 1 — Supplementary material 1 (DOCX 696 kb) [file 10654_2019_593_MOESM1_ESM.docx]

# **Supplementary Materials**

## **Supplementary Table 1. Search terms**

| PubMed | Embase |
| --- | --- |
| (“blood pressure” OR blood pressure[MeSH] OR hypertension OR hypertension[MeSH]) AND (“cardiac arrest” OR cardiac arrest[MeSH] OR “sudden cardiac death” OR sudden cardiac death[MeSH]) AND (“case-control” OR cohort OR cohorts OR prospective OR longitudinal OR “followed up” OR “follow-up” OR “cross-sectional” OR “hazard ratio” OR “relative risk” OR “odds ratio” OR “odds ratios” OR prevalence OR incidence) | (blood pressure OR hypertension OR blood pressure/ OR hypertension/) AND (cardiac arrest OR sudden cardiac death OR cardiac arrest/ OR sudden cardiac death/) AND (case-control OR cohort OR cohorts OR prospective OR longitudinal OR followed up OR follow-up OR cross-sectional OR hazard ratio OR relative risk OR odds ratio OR prevalence OR incidence). |

**Supplementary Table 2. Cohort studies of hypertension and the risk of sudden cardiac death**

| First author, publication year, country | Study name or description | Study period | Number of participants, number of cases | Exposure, subgroup | Comparison | Relative risk (95% confidence interval) | Adjustment for confounders |
| --- | --- | --- | --- | --- | --- | --- | --- |
| Shaper AG et al., 1993, the United Kingdom^29^ | British Regional Heart Study (BRHS) | 1978–1980­–NA, 8 years follow-up | 5813 men without prior CHD, age 40-59 years: 44 SCDs | Hypertension | Yes vs. no | 2.4 (1.2-4.9) | Age, social class, smoking, heavy drinking, physical activity, blood cholesterol, blood glucose |
| Weijenberg MP et al., 1996, the Netherlands^30^ | Zutphen Elderly Study | 1985­–1990, 5 years follow-up | 885 men, age 64-84 years: 44 SCDs | Antihypertensive use       Borderline isolated systolic hypertension^b^ (140 mmHg ≤ SBP < 160 mmHg)  Definite isolated systolic hypertension^b^ (SBP ≥160 mmHg) | Yes vs. no  (and DBP <75 mmHg)  Yes vs. no  (and SBP <140 mmHg)^a^  Yes vs. no      Yes vs. no | 4.32 (1.01-18.40)    8.70 (2.11-35.90)    4.23 (0.86-20.76)  9.20 (1.76-47.97) | Age, BMI, serum total and high-density lipoprotein cholesterol, cigarette smoking, alcohol consumption, use of medications known to lower blood pressure levels without an indication for hypertension, physician who measured the blood pressure |
| Albert CM et al., 2003, the United States^28^ | Nurses’ Health Study (NHS) | 1976­–1998, 22 years follow-up | 121701 female registered nurses, age 30-55 years: 244 SCDs | Hypertension  Hypertension, age ≤60 years^b^  Hypertension, age >60 years^b^ | Yes vs. no  Yes vs. no  Yes vs. no | 2.49 (1.87-3.32)  1.94 (1.26-2.98)  3.07 (2.06-4.57) | Age, diabetes, smoking, hypercholesterolemia, parental history of MI before age 60 years, parental history of MI age ≥60 years, BMI, menopausal status, postmenopausal hormone use, prior report of CHD |
| Straus SMJM et al., 2006, the Netherlands^31^ | Rotterdam Study | 1990–1993–2000, 6.7 years follow-up | 6134 men and women, age ≥55 years: 125 SCDs | Hypertension | Yes vs. no | 2.4 (1.7-3.5) | Age, gender |
| First author, publication year, country | **Study name or description** | **Study period** | **Number of participants, number of cases** | **Exposure, subgroup** | **Comparison** | **Relative risk (95% confidence interval)** | **Adjustment for confounders** |
| Bertoia ML et al., 2012, the United States^6^ | Women’s Health Initiative (WHI) | 1993–1998–2009, 10.8 years follow-up | 160291 post-menopausal women, age 50-79 years: 418 SCDs | Hypertension  Hypertension, without prior CHD^c^ | Yes vs. no  Yes vs. no | 1.46 (1.11-1.93)  1.55 (1.12-2.14) | Age, race, total family income, smoking, resting pulse, BMI, waist-to-hip ratio, WBC, CHD (excluding MI), MI, heart failure, self-reported atrial fibrillation, diabetes mellitus, carotid artery disease |
| Ohira T et al., 2012, Japan^11^ | Circulatory Risk in Communities Study (CIRCS) | 1975/1981–2005, 3.5 years follow-up | 26870 men and women, age 30-84 years: nested case-control study: 239 SCD cases, 717 controls | Hypertension | Yes vs. no | 1.52 (1.05-2.19) | Age, sex, examination year, follow-up time, study area, BMI, excess ethanol intake, diabetes mellitus, current smoking, heart rate, atrial fibrillation, SVPC/VPC, major ST-T abnormalities, minor ST-T abnormalities, wide QRS, left high amplitude R wave, abnormal Q wave |
| Karppi J et al., 2013, Finland^13^ | Kuopio Ischemic Heart Disease Risk Factor Study (KIHD) | 1991–1993–2008, 15.9 years follow-up | 1031 men, age 46-64 years: 59 SCDs | Drug for hypertension | Yes vs. no | 3.82 (2.22-6.56) | Age, SBP, waist circumference, current smoking, alcohol intake, years of education, prevalent CHD, congestive heart failure, diabetes, previous MI, serum 𝛃-carotene, serum low-density lipoprotein cholesterol, serum triglycerides, serum hs-CRP |
| Rapsomaniki E et al., 2014, the United Kingdom^34,d^ | CALIBER programme | 1997–2010–NA, 5.2 years follow-up | 1258006 men and women free of baseline CVDs, age ≥ 30 years: 2355 cardiac arrest/SCD cases | Hypertension | Yes vs. no | 1.20 (1.15-1.25) | Age, competing risk of other cardiovascular diseases and non-cardiovascular disease mortality |
| First author, publication year, country | **Study name or description** | **Study period** | **Number of participants, number of cases** | **Exposure, subgroup** | **Comparison** | **Relative risk (95% confidence interval)** | **Adjustment for confounders** |
| Mannsverk J et al., 2016, Norway^33^ | Tromsø Study | 1994–1995/2001–2002/2007–2008–2010, 12.7 years follow-up | 29582 men and women, age ≥25 years: 332 out-of-hospital sudden deaths | Hypertension | Yes vs. no | 1.75 (1.33-2.29) | Age, sex |
| Waks JW et al., 2016, the United States^12^ | Atherosclerosis Risk in Communities study (ARIC), Cardiovascular Health Study (CHS) | ARIC 1987–1989–2001, 14.1 years follow-up; CHS 1989–1990/1992–1993–2006, 13.1 years follow-up; combined 14.0 years follow-up | ARIC 14609 men and women, age 45-64 years: 291 SCDs; CHS 5568 men and women, age 65-100 years: 195 SCDs; combined 20177 men and women, age 45-100 years: 486 SCDs | Hypertension | Yes vs. no | 2.329 (1.439-3.769) | Age, sex, race, diabetes mellitus, CHD, stroke, SQRT SVG elevation, QRS-T angle, SAI QRST, SAI QRST*age, QRS-T angle*age, QRS-T angle*race, QRS-T angle*diabetes mellitus, QRS-T angle*hypertension, SQRT SVG azimuth*sex |

NA, not available; CHD, coronary heart disease; SCD, sudden cardiac death; DBP, diastolic blood pressure; SBP, systolic blood pressure; BMI, body mass index; MI, myocardial infarction; WBC, white blood cell count; SVPC/VPC, supraventricular premature contraction/ventricular premature contraction; ST-T, ST-segment and T-wave; hs-CRP, high sensitivity C-reactive protein; SVG, spatial ventricular gradient; SAI QRST, sum absolute QRST integral. ^a^ Excluded from the meta-analysis considering the extreme relative risk & the very large range of the confidence interval. ^b^ Unique exposures/subgroups excluded from the meta-analysis. ^c^ Subgroup estimates excluded from the primary analysis and used in subgroup and sensitivity analyses. ^d^ Study only included in sensitivity analysis as it assessed a combined outcome of sudden cardiac arrest/sudden cardiac death.

**Supplementary Table 3. Cohort studies of blood pressure and the risk of sudden cardiac death**

| First author, publication year, country | Study name or description | Study period | Number of participants, number of cases | Exposure, subgroup | Comparison | Relative risk  (95% confidence interval) | Adjustment for confounders |
| --- | --- | --- | --- | --- | --- | --- | --- |
| Kannel WB et al., 1975, the United States^15^ | Albany Study & Framingham Heart Study | Albany 1953–NA, Framingham 1948–1952–NA, 16 years follow-up | 4120 men free of baseline CHD: Albany – 1838 civil servants, age 39-55 years, Framingham – 2282, age 30-62 years: 109 sudden deaths | SBP | Per mmHg | 1.0126 (1.0039-1.0213) | Age, ECG-LVH, metropolitan relative weight, cigarettes/day, cholesterol |
| Schatzkin A et al., 1984, the United States^27,a^ | Framingham Heart Study | 1948­–1952–NA, 26 years follow-up | 2336 men and 2873 women (total 5209) with & without prior CHD, age 30-62 years: 103 sudden unexpected deaths (69 men 34 women) among those without prior CHD | SBP, male, without prior CHD  SBP, female, without prior CHD | Per mmHg    Per mmHg | 1.0106 (1.0004-1.0209)   1.0019 (0.9879-1.0161) | Age, cigarettes, glucose, cholesterol, Framingham relative weight, haematocrit, vital capacity, LVH-ECG, cholesterol*age interaction (male only) |
| Wannamethee G et al., 1995, the United Kingdom^16,b^ | British Regional Heart Study (BRHS) | 1978–1980­–NA, 8 years follow-up | 7735 men, age 40-59 years: 117 SCDs | SBP | ≥161 vs. <128 mmHg | 2.2 (1.2-3.9) | Age |
| Weijenberg MP et al., 1996, the Netherlands^30^ | Zutphen Elderly Study | 1985­–1990, 5 years follow-up | 885 men, age 64-84 years: 44 SCDs | SBP      DBP | <140 mmHg  140-159 mmHg  ≥160 mmHg  <75 mmHg  75-84 mmHg  85-94 mmHg  ≥95 mmHg | 1.00  4.80 (1.36-16.95)  5.56 (1.54-20.08)  1.00  1.42 (0.37-5.51)  2.03 (0.53-7.73)  3.04 (0.75-12.37) | Age, BMI, serum total and high-density lipoprotein cholesterol, cigarette smoking, alcohol consumption, use of medications known to lower blood pressure levels without an indication for hypertension, physician who measured the blood pressure |
| First author, publication year, country | **Study name or description** | **Study period** | **Number of participants, number of cases** | **Exposure, subgroup** | **Comparison** | **Relative risk  (95% confidence interval)** | **Adjustment for confounders** |
| Jouven X et al., 2001, France^17^ | Paris Prospective Study 1 | 1967–1972–1994, 23 years follow-up | 7079 male civil servants free of baseline CHD, age 42-53 years: 118 sudden deaths | SBP | Per 20 mmHg | 1.21 (1.06-1.37) | Age, parental sudden death, parental MI, sport activity, diabetic status, tobacco consumption, BMI, resting heart rate, total cholesterol |
| Lahtinen AM et al., 2012, Finland^18^ | FINRISK 1992 | 1992­–2008, 16.8 years follow-up | 5345 men and women, age 25-74 years: 129 SCDs | SBP | Per 10 mmHg | 1.17 (1.08-1.26) | Age, sex, geographic region, HDL/TC ratio, prevalent diabetes, BMI, current smoker, former smoker, physical activity, prevalent CHD |
| Lahtinen AM et al., 2012, Finland^18^ | FINRISK 1997 | 1997–2008, 11.8 years follow-up | 7672 men and women, age 25-74 years: 178 SCDs | SBP | Per 10 mmHg | 1.06 (0.98-1.13) | Age, sex, geographic region, HDL/TC ratio, prevalent diabetes, BMI, current smoker, former smoker, physical activity, prevalent CHD, QT-prolonging drug, Digoxin |
| Lahtinen AM et al., 2012, Finland^18^ | FINRISK 2002 | 2002–2008, 6.8 years follow-up | 8212 men and women, age 25-74 years: 75 SCDs | SBP | Per 10 mmHg | 1.12 (1.02-1.24) | Age, sex, geographic region, HDL/TC ratio, prevalent diabetes, BMI, current smoker, former smoker, physical activity, prevalent CHD, QT-prolonging drug, Digoxin |
| Lahtinen AM et al., 2012, Finland^18^ | Health 2000 | 2000–2001–2008, 8.0 years follow-up | 6400 men and women, age ≥30 years: 112 SCDs | SBP | Per 10 mmHg | 1.06 (0.97-1.16) | Age, sex, geographic region, HDL/TC ratio, prevalent diabetes, BMI, current smoker, former smoker, physical activity, prevalent CHD, QT-prolonging drug, Digoxin |
| First author, publication year, country | **Study name or description** | **Study period** | **Number of participants, number of cases** | **Exposure, subgroup** | **Comparison** | **Relative risk  (95% confidence interval)** | **Adjustment for confounders** |
| Laukkanen JA et al., 2012, Finland^32,c^ | Kuopio Ischemic Heart Disease Risk Factor Study (KIHD) | 1984–1989–2008, 18.9 years follow-up | 2666 men, age 42-61 years: 213 SCDs | SBP  DBP | Per 10 mmHg  Per 10 mmHg | 1.15 (1.07-1.25)  1.17 (1.02-1.35) | Age, alcohol consumption, cigarette smoking, serum low-density lipoprotein cholesterol, type 2 diabetes, BMI, left ventricular hypertrophy, previous MI, family history of CHD, use of antihypertensive medications |
| Laukkanen JA et al., 2012, Finland^32,d^ | Kuopio Ischemic Heart Disease Risk Factor Study (KIHD) | 1984–1989–2008, 18.9 years follow-up | 2666 men, age 42-61 years: 213 SCDs | SBP  DBP | <123 mmHg  123-132 mmHg  133-145 mmHg  >145 mmHg  <82 mmHg  82-88 mmHg  88-95 mmHg  >95 mmHg | 1.00  0.96 (0.61-1.50)  1.25 (0.82-1.92)  2.04 (1.37-3.03)  1.00  1.03 (0.68-1.56)  1.24 (0.82-1.85)  1.60 (1.06-2.41) | Age, alcohol consumption, cigarette smoking, serum low-density lipoprotein cholesterol, type 2 diabetes, BMI, left ventricular hypertrophy, previous MI, family history of CHD, use of antihypertensive medications |
| Rapsomaniki E et al., 2014, the United Kingdom^34,c,e^ | CALIBER programme | 1997–2010–NA, 5.2 years follow-up | 1258006 men and women free of baseline CVDs, age ≥ 30 years: 2355 cardiac arrest/SCD cases | SBP  DBP | Per 20 mmHg  Per 10 mmHg | 1.08 (0.99-1.18)  1.11 (1.03-1.21) | Age, sex, primary care practice, smoking, diabetes, total and high-density lipoprotein cholesterol, BMI, blood pressure lowering medications |
| First author, publication year, country | **Study name or description** | **Study period** | **Number of participants, number of cases** | **Exposure, subgroup** | **Comparison** | **Relative risk  (95% confidence interval)** | **Adjustment for confounders** |
| Rapsomaniki E et al., 2014, the United Kingdom^34,d,e^ | CALIBER programme | 1997–2010–NA, 5.2 years follow-up | 1258006 men and women free of baseline CVDs, age ≥ 30 years: 2355 cardiac arrest/SCD cases | SBP            DBP | 90-114 mmHg  115-129 mmHg  130-139 mmHg  140-159 mmHg  160-179 mmHg  ≥180 mmHg  60-74 mmHg  75-84 mmHg  85-89 mmHg  90-94 mmHg  95-99 mmHg  ≥100 mmHg | - 1. (0.87-1.18)   1.00 (0.94-1.06)  1.08 (0.94-1.26)  1.37 (1.15-1.64)  1.67 (1.33-2.10)  2.05 (1.51-2.78)  0.96 (0.81-1.13)  1.04 (1.00-1.08)  1.22 (1.12-1.34)  1.41 (1.24-1.60)  1.63 (1.35-1.96)  2.03 (1.54-2.68) | Age, sex, primary care practice, smoking, diabetes, total and high-density lipoprotein cholesterol, BMI, blood pressure lowering medications |
| Bogle BM et al., 2018, the United States^7^ | Atherosclerosis Risk in Communities study (ARIC - derivation) | ARIC 1987–1989–NA, 10 years follow-up | 11335 white men and women, age 45-64 years: 95 SCDs  3780 black men and women, age 45-64 years: 50 SCDs | SBP, white^f^ DBP, white^f^ SBP, black^f^ DBP, black^f^ | Per mmHg  Per mmHg  Per mmHg  Per mmHg | 1.025 (1.016-1.035)  0.976 (0.957-0.996)  1.017 (1.005-1.029)  0.995 (0.973-1.017) | Age, sex, total cholesterol, lipid-lowering medication use, hypertension medication use, current smoker, diabetes, BMI |

NA, not available; CHD, coronary heart disease; SBP, systolic blood pressure; ECG-LVH, electrocardiographic evidence of left ventricular hypertrophy; SCD, sudden cardiac death; DBP, diastolic blood pressure; BMI, body mass index; MI, myocardial infarction; HDL/TC ratio, high-density lipoprotein-total cholesterol concentration ratio. ^a^ Study only included in subgroup analyses by sex. ^b^ Study excluded from the meta-analysis due to inadequate data (insufficient blood pressure categories for data analysis). ^c^ Data used for linear dose-response analysis. ^d^ Data used for nonlinear dose-response analysis. ^e^ Study only included in sensitivity analysis as it assessed a combined outcome of sudden cardiac arrest/sudden cardiac death. ^f^ Race-specific estimates pooled using the fixed effect model before proceeding to the meta-analysis.

## **Supplementary Table 4. Relative risks and 95% confidence intervals from the nonlinear dose-response analysis of cohort studies on systolic blood pressure and the risk of sudden cardiac death**

| Systolic Blood Pressure (mmHg) | Relative Risks (95% Confidence Intervals) |
| --- | --- |
| 118 | 1.00 |
| 120 | 1.01 (0.91-1.11) |
| 125 | 1.05 (0.84-1.33) |
| 130 | 1.15 (0.84-1.57) |
| 135 | 1.28 (0.90-1.84) |
| 140 | 1.46 (1.01-2.12) |
| 145 | 1.71 (1.18-2.48) |
| 150 | 2.00 (1.38-2.91) |
| 155 | 2.39 (1.62-3.53) |
| 160 | 2.87 (1.85-4.44) |
| 165 | 3.43 (2.07-5.70) |
| 170 | 4.15 (2.27-7.59) |
| 175 | 4.99 (2.44-10.18) |
| 180 | 6.04 (2.60-14.03) |

## **Supplementary Table 5. Relative risks and 95% confidence intervals from the nonlinear dose-response analysis of cohort studies on diastolic blood pressure and the risk of sudden cardiac death**

| Diastolic Blood Pressure (mmHg) | Relative Risks (95% Confidence Intervals) |
| --- | --- |
| 70 | 1.00 |
| 75 | 1.07 (0.73-1.57) |
| 80 | 1.16 (0.61-2.21) |
| 85 | 1.29 (0.57-2.93) |
| 90 | 1.47 (0.61-3.56) |
| 95 | 1.73 (0.74-4.01) |
| 100 | 2.09 (1.01-4.29) |
| 105 | 2.60 (1.39-4.89) |
| 110 | 3.39 (1.44-7.98) |
| 115 | 4.54 (1.06-19.51) |
| 120 | 6.34 (0.62-65.29) |

## **Supplementary Table 6. Quality Assessment of Individual Studies using the Newcastle-Ottawa Quality Assessment Scale**

| Study | Selection | Comparability | Outcome | Total Score |
| --- | --- | --- | --- | --- |
| Kannel WB et al., 1975 | ✕☆☆☆ | ☆☆ | ☆☆☆ | **8** |
| Schatzkin A et al., 1984 | ☆☆☆☆ | ☆☆ | ☆☆☆ | **9** |
| Shaper AG et al., 1993 | ☆☆☆☆ | ☆☆ | ☆☆✕ | **8** |
| Wannamethee G et al., 1995 | ☆☆☆✕ | ☆✕ | ☆☆☆ | **7** |
| Weijenberg MP et al., 1996 | ☆☆☆✕ | ☆☆ | ☆☆☆ | **8** |
| Jouven X et al., 2001 | ✕☆☆☆ | ☆☆ | ☆☆☆ | **8** |
| Albert CM et al., 2003 | ✕☆✕✕ | ☆☆ | ☆☆☆ | **6** |
| Straus SMJM et al., 2006 | ☆☆☆✕ | ☆☆ | ☆☆☆ | **8** |
| Bertoia ML et al., 2012 | ☆☆☆✕ | ☆☆ | ☆☆✕ | **7** |
| Lahtinen AM et al., 2012 | ☆☆☆✕ | ☆☆ | ☆☆☆ | **8** |
| Lahtinen AM et al., 2012 | ☆☆☆✕ | ☆☆ | ☆☆☆ | **8** |
| Lahtinen AM et al., 2012 | ☆☆☆✕ | ☆☆ | ☆☆☆ | **8** |
| Lahtinen AM et al., 2012 | ☆☆☆✕ | ☆☆ | ☆☆☆ | **8** |
| Laukkanen et al., 2012 | ✕☆☆✕ | ☆☆ | ☆☆☆ | **7** |
| Ohira T et al., 2012 | ☆☆☆✕ | ☆☆ | ☆✕✕ | **6** |
| Karppi J et al., 2013 | ☆☆☆✕ | ☆☆ | ☆☆☆ | **8** |
| Rapsomaniki E et al., 2014 | ☆☆☆☆ | ☆☆ | ☆☆✕ | **8** |
| Mannsverk J et al., 2016 | ☆☆☆☆ | ☆☆ | ☆☆☆ | **9** |
| Waks JW et al., 2016 | ☆☆☆✕ | ☆☆ | ☆☆✕ | **7** |
| Khosravi A et al., 2017 | ☆☆☆☆ | ☆☆ | ☆☆✕ | **8** |
| Bogle BM et al., 2018 | ☆☆☆✕ | ☆☆ | ☆☆✕ | **7** |

## **Supplementary Fig. 1 Funnel plot of the meta-analysis of cohort studies on hypertension and sudden cardiac death**

## **Supplementary Fig. 2 Influence analysis excluding one study at a time for the meta-analysis of cohort studies on hypertension and sudden cardiac death**

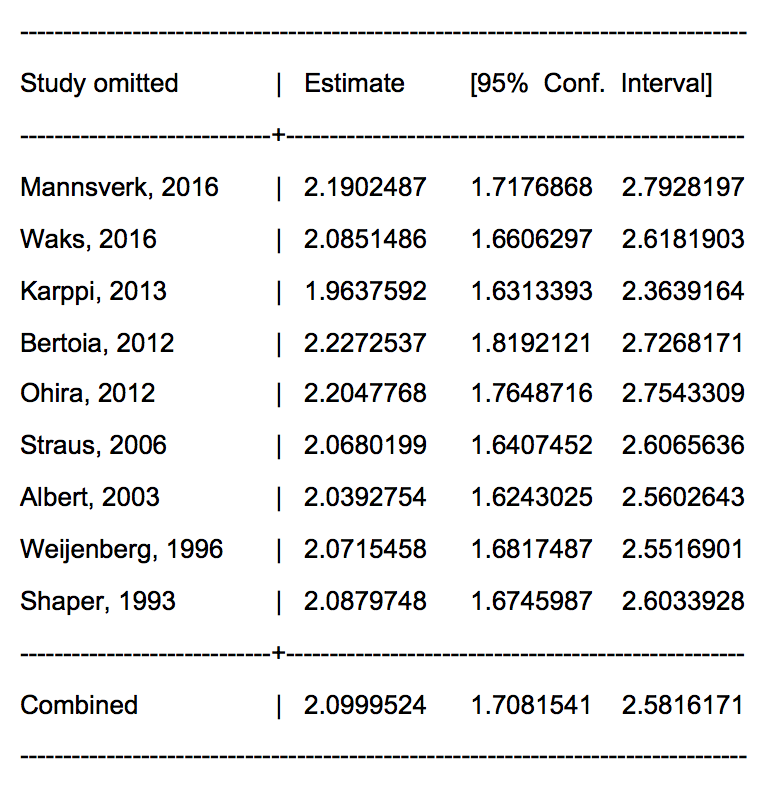


## **Supplementary Fig. 3 Funnel plot of the meta-analysis of cohort studies on systolic blood pressure and sudden cardiac death**

## **Supplementary Fig. 4 Influence analysis excluding one study at a time for the meta-analysis of cohort studies on systolic blood pressure (per 20 mmHg) and sudden cardiac death**

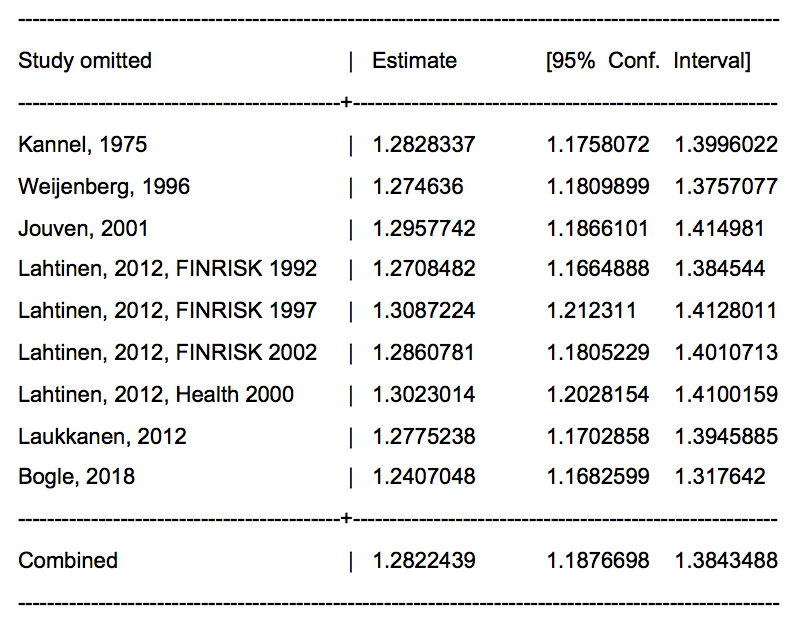


## **Supplementary Fig. 5 Funnel plot of the meta-analysis of cohort studies on diastolic blood pressure and sudden cardiac death**
